# Supplementary material for: Steroid Pulse Therapy Leads to Secondary Infections and Poor Outcomes in Patients with Severe Acute Respiratory Syndrome Coronavirus 2 (SARS-CoV-2) in Intensive Care Units: A Retrospective Cohort Study
Source: Viruses. 2025 Jun 6;17(6):822. doi: 10.3390/v17060822 (PMC12197673; doi:10.3390/v17060822)
Supplement: Supplementary file 1 [file viruses-17-00822-s001.zip › R1 Supplementary files250524/Table S3Total steroid dose and duration ofü@steroidü@therapy250524JY.docx]

Table S3　Total steroid dose and duration of　steroid　therapy　between　non-secondary　infection　group　and　secondary　infection　group.

|  | Non-Secondary infection group (n = 55) | Secondary infection group (n = 21) | P-value* |
| --- | --- | --- | --- |
| Total steroid dose (mg) | 4275.0 (1144.5-4582.5) | 1331.3 (336.9-4536.3) | 0.0591 |
| Duration of steroid therapy (days) | 20.0 (13.0-25.0) | 18.0 (9.0-24.0) | 0.1810 |

Non-parametric continuous variables were presented as medians and interquartile ranges (IQR; first quartile and third quartile) and were compared using the Wilcoxon signed-rank test.

* We determined the significance level to be 5%.
