# Supplementary material for: Steroid Pulse Therapy Leads to Secondary Infections and Poor Outcomes in Patients with Severe Acute Respiratory Syndrome Coronavirus 2 (SARS-CoV-2) in Intensive Care Units: A Retrospective Cohort Study
Source: Viruses. 2025 Jun 6;17(6):822. doi: 10.3390/v17060822 (PMC12197673; doi:10.3390/v17060822)
Supplement: Supplementary file 1 [file viruses-17-00822-s001.zip › R1 Supplementary files250524/Table S4Multicollinearity 250524JY.docx]

**Table S4.** Multicollinearity Diagnostics Table

| **Variable** | **p-value** | **Tolerance** | **VIF^＊^** |
| --- | --- | --- | --- |
| Age | .003 | 0.895 | 1.117 |
| Remdesivir | .008 | 0.875 | 1.143 |
| Pulse therapy | <.001 | 0.929 | 1.077 |

＊VIF: Variance Inflation Factor
