# Supplementary material for: Steroid Pulse Therapy Leads to Secondary Infections and Poor Outcomes in Patients with Severe Acute Respiratory Syndrome Coronavirus 2 (SARS-CoV-2) in Intensive Care Units: A Retrospective Cohort Study
Source: Viruses. 2025 Jun 6;17(6):822. doi: 10.3390/v17060822 (PMC12197673; doi:10.3390/v17060822)
Supplement: Supplementary file 1 [file viruses-17-00822-s001.zip › R1 Supplementary files250524/TableS1CONUTscore250524.docx]

TableS1 CONUT score

Table S2 was made referred to González-Madroño, A.; Mancha, A.; Rodríguez, F.J.; Culebras, J.; de Ulibarri, J.I. Confirming the validity of the CONUT system for early detection and monitoring of clinical undernutrition: comparison with two logistic regression models developed using SGA as the gold standard. Nutr Hosp 2012, 27, 564–571. DOI:10.1590/S0212-16112012000200033.
