# Supplementary material for: Steroid Pulse Therapy Leads to Secondary Infections and Poor Outcomes in Patients with Severe Acute Respiratory Syndrome Coronavirus 2 (SARS-CoV-2) in Intensive Care Units: A Retrospective Cohort Study
Source: Viruses. 2025 Jun 6;17(6):822. doi: 10.3390/v17060822 (PMC12197673; doi:10.3390/v17060822)
Supplement: Supplementary file 1 [file viruses-17-00822-s001.zip › R1 Supplementary files250524/TableS2 Secondary Infection250524JY.docx]

Table S2　Comparison of occurrence of secondary Infection and infection focus between non-survivors and survivors

|  | Non-survivors  (n = 23) | Survivors  (n = 53) | Odds ratio | 95 % CI | P-value** |
| --- | --- | --- | --- | --- | --- |
| Secondary Infection (n, %) | 22 (95.7) | 29 (54.7) | 0.0550 | 0.0069-0.4378 | 0.0005 |
| Pneumonia (n, %)* | 20 (87.0) | 26 (49.1) | 0.1444 | 0.0383-0.5449 | 0.0019 |
| Urinary Tract Infection (n, %) | 10 (43.5) | 10 (18.9) | 0.3023 | 0.1033-0.8848 | 0.0252 |
| Bacteremia (n, %) | 10 (43.5) | 11 (20.8) | 0.3405 | 0.1181-0.9812 | 0.0418 |

*Pneumonia were excluded in COVID-19 pneumonia

**Categorical variables were presented as numbers (n) and percentages (%)

Chi-square tests or Fisher’s exact probability tests were performed for categorical variables.

We determined the optimal cutoff points and significance level to be 5%.
