# Supplementary figures and images for: Steroid Pulse Therapy Leads to Secondary Infections and Poor Outcomes in Patients with Severe Acute Respiratory Syndrome Coronavirus 2 (SARS-CoV-2) in Intensive Care Units: A Retrospective Cohort Study
Source: Viruses. 2025 Jun 6;17(6):822. doi: 10.3390/v17060822 (PMC12197673; doi:10.3390/v17060822)

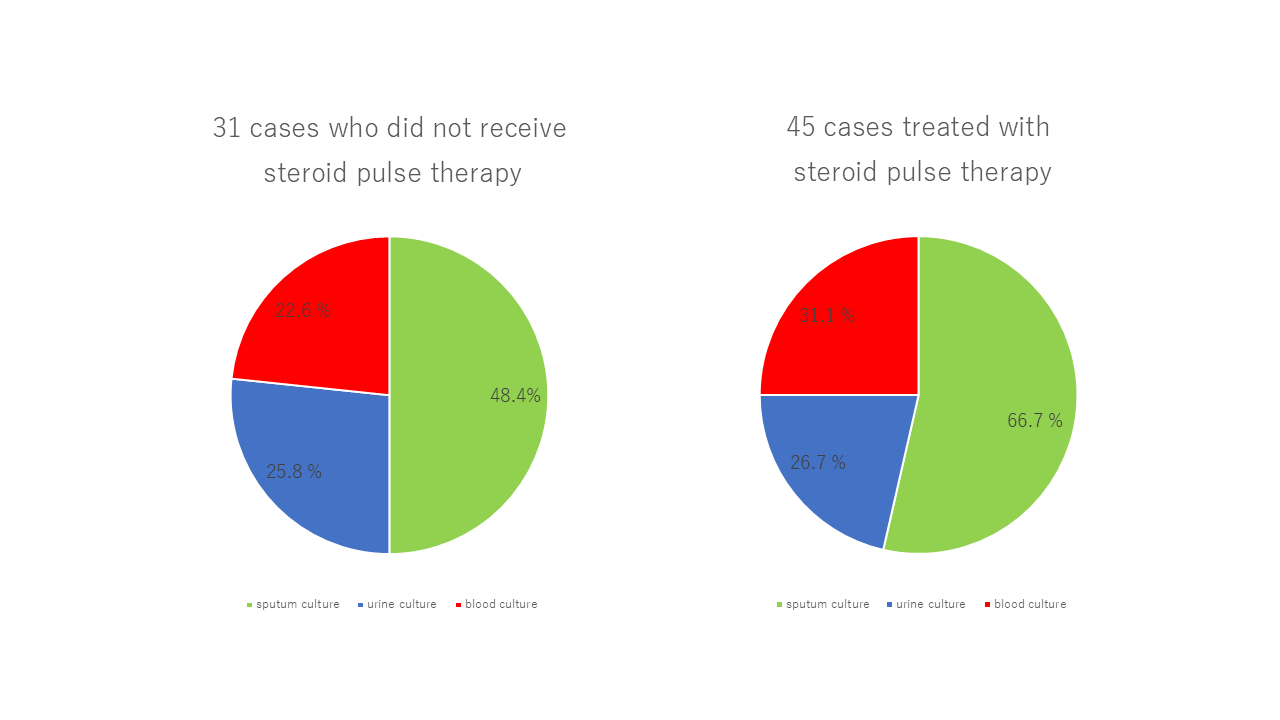

Supplement: Supplementary file 1 [file viruses-17-00822-s001.zip › Figures files250606/Figure 2The pathogen of secondary infection in this study250524.tif]

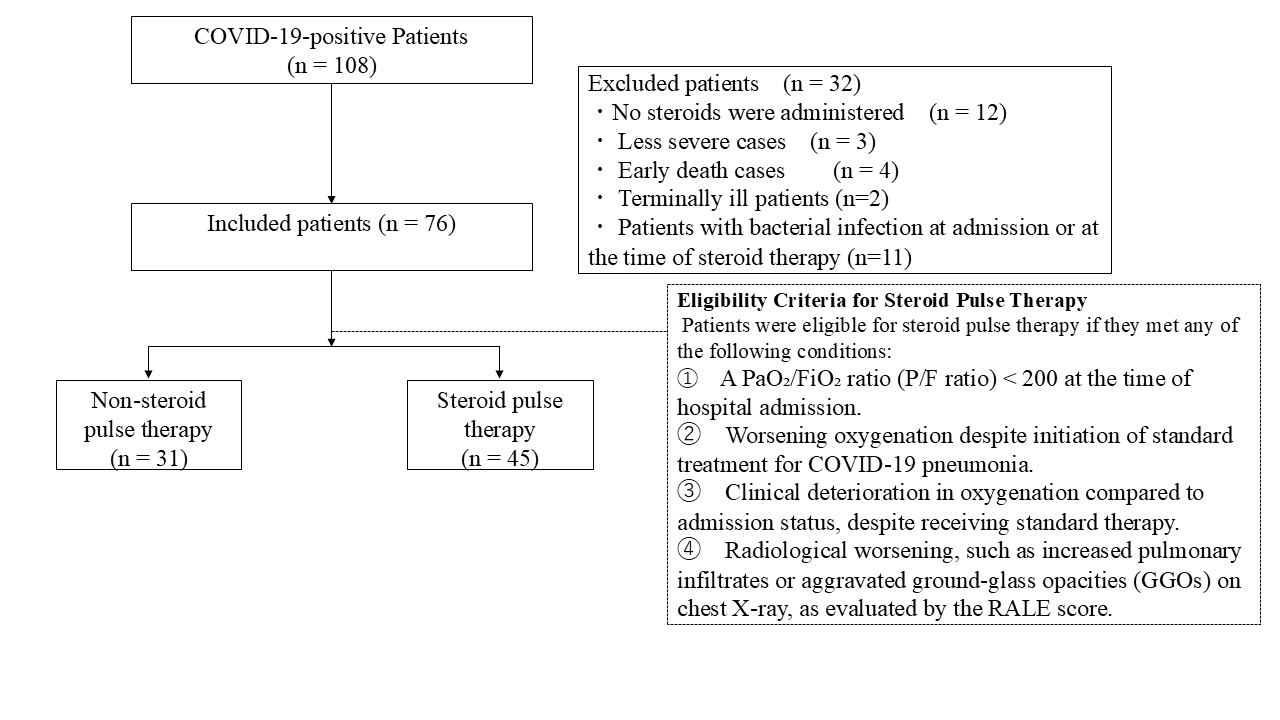

Supplement: Supplementary file 1 [file viruses-17-00822-s001.zip › Figures files250606/Figure1-250606.tif]

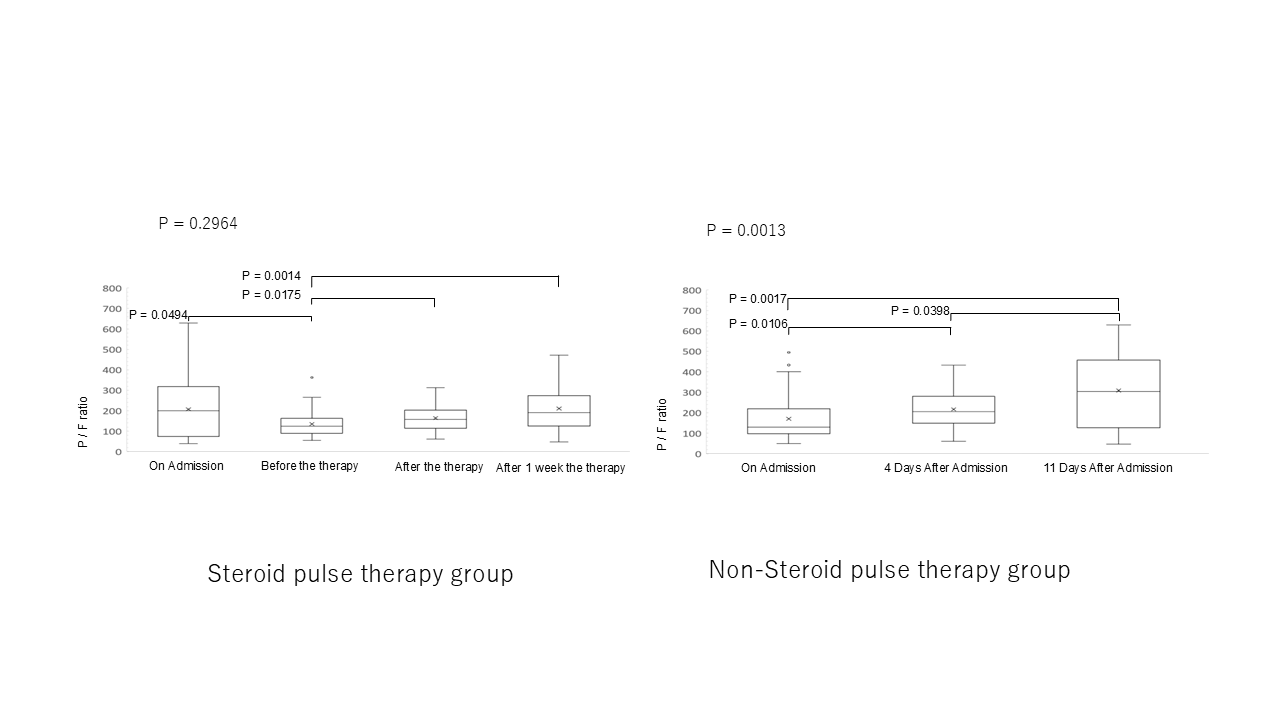

Supplement: Supplementary file 1 [file viruses-17-00822-s001.zip › R1 Supplementary files250524/Figure S2 Comparison of PFratio overtime 250524JY.tif]

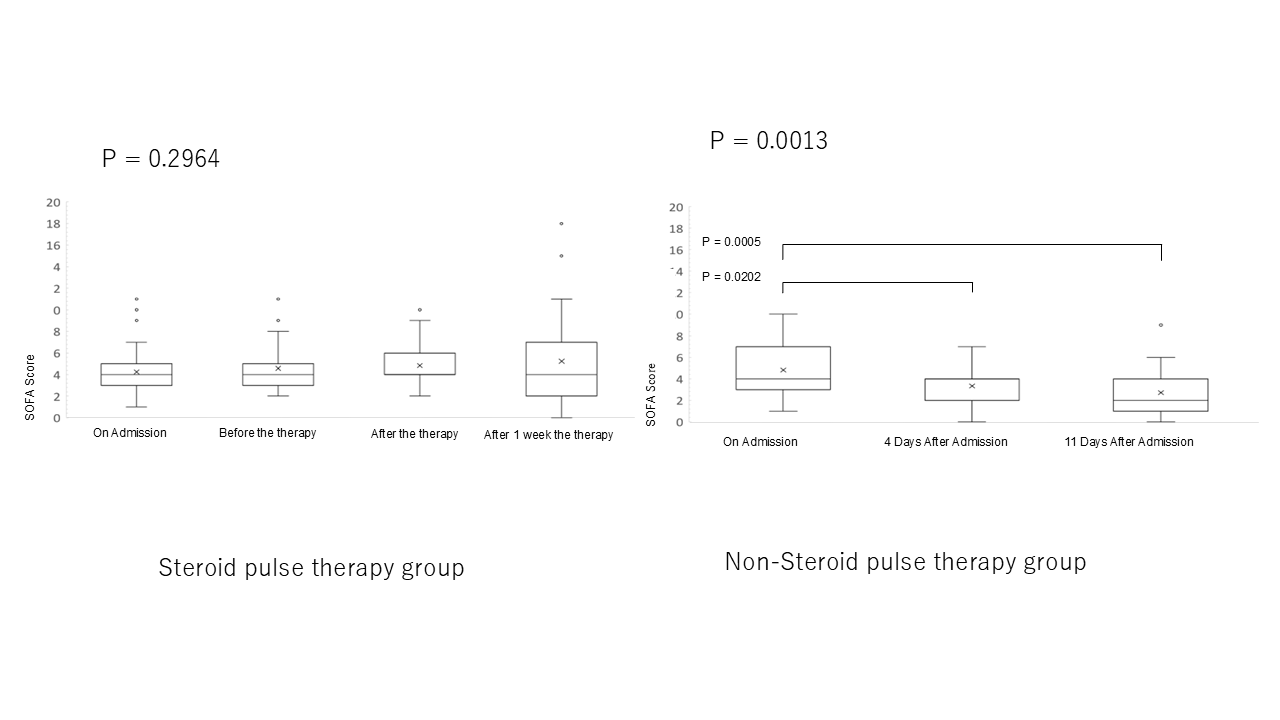

Supplement: Supplementary file 1 [file viruses-17-00822-s001.zip › R1 Supplementary files250524/Figure S3Comparison of SOFA score overtime 250524JY.tif]

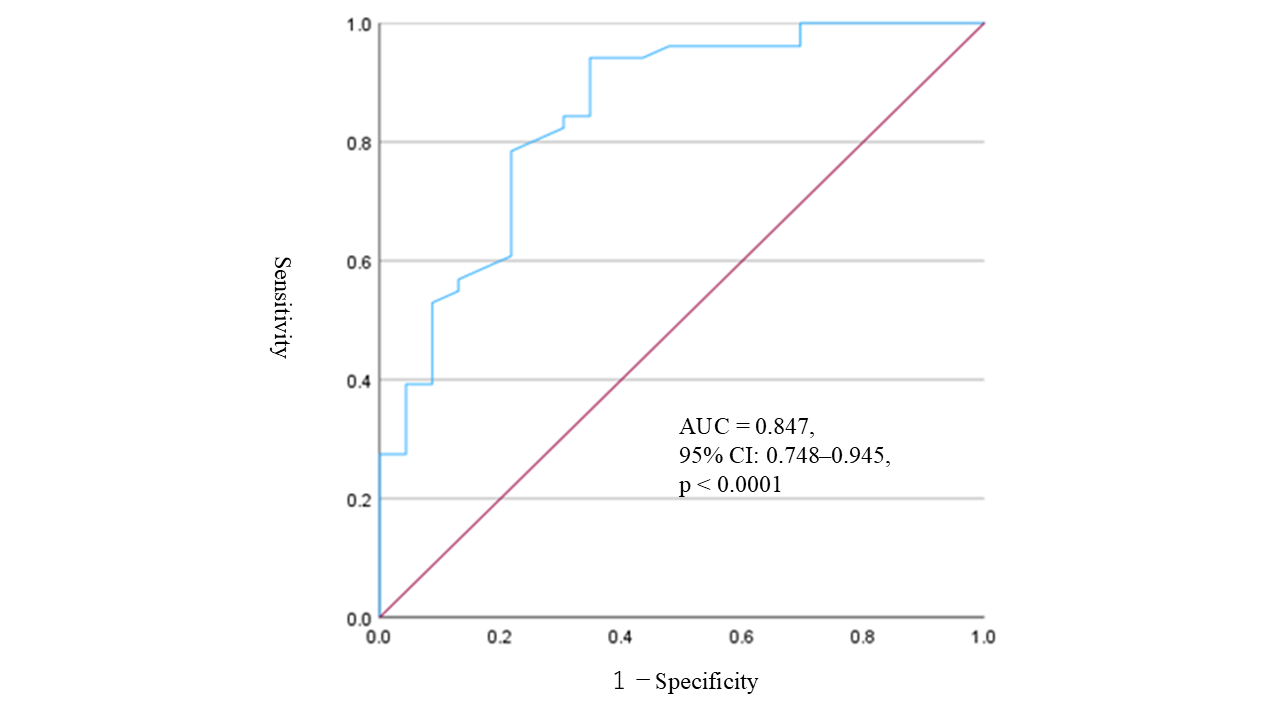

Supplement: Supplementary file 1 [file viruses-17-00822-s001.zip › R1 Supplementary files250524/FIgureS1 Model prediction based on ROC curve250524JY.tif]

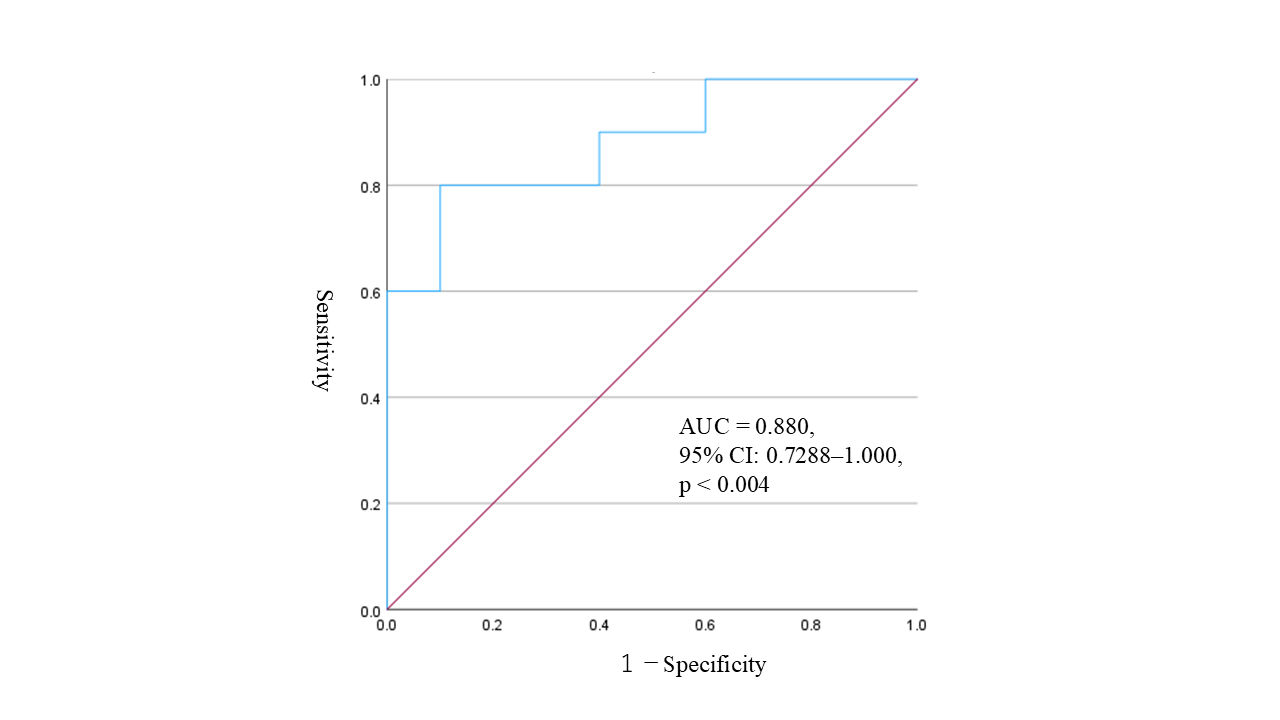

Supplement: Supplementary file 1 [file viruses-17-00822-s001.zip › R1 Supplementary files250524/FigureS4 ROCcurve based on the developed using the training cohort250524JY.tif]
